# Plasma 25-Hydroxyvitamin D Levels and VDR Gene Expression in Peripheral Blood Mononuclear Cells of Leukemia Patients and Healthy Subjects in Central Kazakhstan

**DOI:** 10.3390/nu12051229

**Published:** 2020-04-26

**Authors:** Assel G. Zhumina, Konstantin Li, Anna A. Konovalova, Yelena A. Li, Margarita Yu. Ishmuratova, Gayane P. Pogossyan, Michael Danilenko

**Affiliations:** 1 Department of Botany, Academician Y.A. Buketov Karaganda State University, Karaganda 100028, Kazakhstan; asbiol@list.ru (A.G.Z.); anjuta.kon_1986@mail.ru (A.A.K.); margarita.ishmur@mail.ru (M.Y.I.); gayane_63@mail.ru (G.P.P.); 2 DNA Diagnostics Laboratory, the Dippner Health Center, Karaganda 100009, Kazakhstan; lee11@mail.ru (K.L.); hellenala@gmail.com (Y.A.L.); 3 Department of Clinical Biochemistry and Pharmacology, Faculty of Health Sciences, Ben-Gurion University of the Negev, Beer-Sheva 84105, Israel

**Keywords:** vitamin D, 25(OH)D, vitamin D receptor, leukemia, observational study

## Abstract

Low blood levels of the vitamin D metabolite 25-hydroxyvitamin D [25(OH)D] have been associated with an increased risk and poorer outcomes of various cancers, including hematological malignancies. The Central Kazakhstan area has a relatively high incidence rate of leukemia. However, the relationship between vitamin D status and leukemia or other types of cancer in Kazakhstan has not yet been addressed. Therefore, in this first pilot single-center study conducted in Central Kazakhstan, we compared plasma levels of 25(OH)D and the vitamin D receptor (VDR) gene expression levels in peripheral blood mononuclear cells of patients with leukemia and demographically matching healthy volunteers. The levels of 25(OH)D in patients were found to be significantly lower (10.8 ± 7.0 ng/mL; *n* = 31) than in healthy subjects (21.6 ± 7.8 ng/mL; *n* = 34; *p* < 0.0001). A similar difference was observed in both younger (<60 years old) and older (>60 years old) participants, though there was no association between 25(OH)D concentration and age within the patient group. In female patients, 25(OH)D levels were significantly lower than in male patients (*p* = 0.04). No significant seasonal variations of 25(OH)D were observed in either the patient or the control group. VDR gene expression levels appeared to be similar in leukemia patients and healthy subjects, and no correlation between the cellular VDR expression and plasma 25(OH)D concentrations was observed in either group of participants. We did not observe a significant association of 25(OH)D or VDR levels and overall survival of leukemia patients. This observational study conducted for the first time in Kazakhstan supports previous findings demonstrating reduced blood 25(OH)D levels in cancer (leukemia) patients. Larger studies are required to determine whether low 25(OH)D plasma concentrations represent a risk factor for leukemia development and/or progression.

## 1. Introduction

Vitamin D_3_ (cholecalciferol) is produced in the epidermis under the influence of UVB and is also consumed from animal foods. In the liver, vitamin D_3_ undergoes 25-hydroxylation to form 25-hydroxyvitamin D_3_ [25(OH)D] followed by an additional 1α-hydroxylation in the kidneys which results in the formation of 1α,25-dihydroxyvitamin D_3_ [1,25(OH)_2_D_3_], the active hormonal form. 1,25(OH)_2_D_3_ has a critical role in maintaining the level of calcium and phosphorus in the blood and regulating many other processes, both in healthy and cancer cells, including proliferation, differentiation, apoptosis, immune responses, and angiogenesis [1,2]. Mechanistically, 1,25(OH)_2_D_3_ exerts most of its biological effects through the activation of the nuclear vitamin D receptor (VDR) transcription factor, which can lead to the activation of more than 1000 genes [3,4].

The vitamin D status in the human body is assessed by measuring blood levels of 25(OH)D, since its serum concentrations are more stable and are less influenced by hormonal mechanisms and by changes in calcium and phosphorus levels, as compared to 1,25(OH)_2_D_3_ [5]. There is still no definite consensus on the optimal vitamin D status, and the recommendations provided by different governmental agencies and scientific societies vary to some extent [6]. For instance, the adequate serum 25(OH)D levels recommended by the Institute of Medicine [7] and the Endocrine Society [8] are above 20 (50 nmol/L) and 30 ng/mL (75 nmol/L), respectively. However, most researchers agree that 25(OH)D levels below 10–12 ng/mL (25–30 nmol/L) represent clinical deficiency, which is considered as a global health problem [9,10,11]. Low circulating levels of 25(OH)D have been associated with an increased risk of colorectal and bladder cancers, while for breast, ovarian and prostate cancers, this association appears to be more controversial [12]. Low serum 25(OH)D levels were also associated with poorer prognosis in patients with hematological malignancies, including leukemia and lymphoma, as well as myeloproliferative/myelodysplastic syndromes [13,14,15]. Several studies have shown that some types of malignant hematopoietic cells highly express VDR, implying their responsiveness to vitamin D-based treatment, which may lead to antitumor effects [14].

To date, there have been only two peer-reviewed publications related to vitamin D status in Kazakhstan. Nugmanova et al. [16] reported fairly adequate serum 25(OH)D levels (median, 24.42 ng/mL; range, 16.22–34.10) in the majority of HIV-infected patients under a medical care in the southern city of Almaty. On the other hand, the most recent cross-sectional study has demonstrated that the majority of adult residents of several geographical areas of the country, including Central Kazakhstan, had insufficient levels of vitamin D (<20 ng/mL) and that the lowest levels (<10 ng/mL) were commonly observed in females [17]. The Central Kazakhstan area has a relatively high incidence rate of leukemia [18]; however, the vitamin D status of patients with leukemia or other types of cancer has not yet been addressed in Kazakhstan. As vitamin D insufficiency may be associated with cancer risk, we hypothesized that in the Central Kazakhstan city of Karaganda and the surrounding area, patients with newly diagnosed leukemias might present even lower circulating 25(OH)D concentrations than healthy residents. Thus, we compared plasma 25(OH)D levels and the expression of VDR in peripheral blood mononuclear cells (PBMCs) of yet untreated adult patients with different types of leukemia and matched healthy volunteers. The major finding of this work was that while there was no significant difference in VDR expression between the two groups of participants, the majority of the patients, particularly females, had a vitamin D deficiency (10.8 ± 7.0 ng/mL) compared to a borderline sufficient vitamin D status (21.6 ± 7.8 ng/mL) in healthy male and female subjects (*p* < 0.0001). 

## 2. Materials and Methods 

### 2.1. Study Subjects

We enrolled 31 adult patients who were admitted to the hematological ward of Karaganda Regional Clinical Hospital (Karaganda, Kazakhstan) with newly diagnosed acute myeloid leukemia (AML), acute lymphoblastic leukemia (ALL), chronic myeloid leukemia (CML) in the chronic phase, and chronic lymphocytic leukemia (CLL). Blood samples for the 25(OH)D and VDR assays (see below) were collected from all enrolled and consented patients prior to beginning of treatment. The demographically matched control group included 34 healthy adult residents of Karaganda and the Karaganda region. Demographic and clinical data of the patients and healthy volunteers are presented in Table 1 and Table 2, respectively. The protocol of the study was approved by the Bioethics Committee of Karaganda Medical University (authorization number 0029; December 23, 2015). All participants gave their written informed consent. 

### 2.2. Blood Samples

Blood samples were collected from patients and healthy volunteers between January 2016 and July 2017. Blood was taken in two sterile evacuated tubes with K_3_-EDTA as an anticoagulant. One tube was used for the preparation of plasma and the other for the isolation of PBMCs.

### 2.3. Plasma 25(OH)D Assay

Within 1 h of collection, blood was centrifuged at room temperature at 4,000 rpm, for 15 min. The plasma was withdrawn and transferred into polypropylene tubes. The 25(OH)D concentration was then measured using the 25OH Vitamin D Total ELISA kit, (DIAsource ImmunoAssays, Louvain-la-Neuve, Belgium) according to the manufacturer’s instructions. 

### 2.4. Isolation of Peripheral Blood Mononuclear Cells (PBMC)

Mononuclear cells were isolated from peripheral blood by density gradient centrifugation using a Ficoll-Paque^TM^ PREMIUM reagent (GE Healthcare Bio-Sciences AB, Uppsala, Sweden), as described previously [19].

### 2.5. Preparation of Total RNA and Quantitative Reverse Transcription Polymerase Chain Reaction (RT-qPCR)

Total RNA was extracted from mononuclear cells using a RIBO-zol-A kit (AmpliSens, Bratislava, Slovak Republic). The RNA concentration and purity were measured spectrophotometrically on a NanoVue Plus instrument (Biochrom, Cambridge, England). An aliquot of 100 ng of mRNA was used as a template to generate cDNA by a REVERTA-L reverse transcriptase kit (AmpliSens, Bratislava, Slovak Republic). The cDNA samples were then diluted 1:25 prior to the PCR reaction. Quantitative cDNA amplification was performed with 5 μl of the diluted cDNA template in a total volume of 20 μl in a DT-322 real-time PCR cycler (DNA Technology, Moscow, Russia) using SYBR Green I dye (Sigma-Aldrich, St. Louis, MO, USA). The primer sequences (Sigma-Aldrich, Rehovot, Israel) were as follows. VDR [NCBI Gene ID 7421] forward primer (5’-GACCTGTGGCAACCAAGACT-3’), reverse primer (5’-AATCAGCTCCAGGCTGTGTC-3’); RPLP0 (reference gene) (NCBI Gene ID 6175) forward primer (5’-AGATGCAGCAGATCCGCAT-3’), reverse primer (5’-GTGGTGATACCTAAAGCCTG-3’). Standard cycling conditions were: 3 min initial enzyme activation at 94 °C then 42 cycles as follows—5 s at 94 °C and 30 s at the annealing temperature (60 °C for both VDR and RPLP0). The calibrator was the cDNA sample of a healthy volunteer with a high plasma 25(OH)D level. The relative expression level was quantified using the 2^-ΔΔCt^ method [20].

### 2.6. Statistical Analysis 

**Figure 1:**
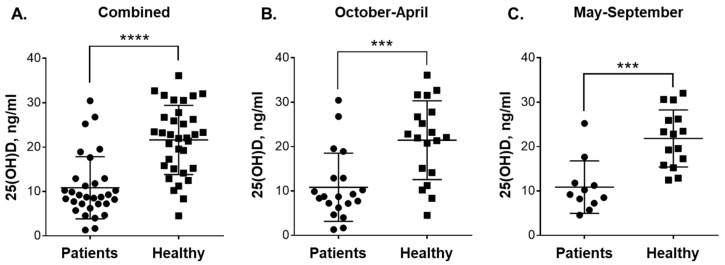
Plasma 25(OH)D levels in blood samples of patients with leukemia and healthy subjects taken during colder and warmer seasons. Comparison between: (**A**) all the participated patients (n = 31) and healthy subjects (*n* = 34); (**B**) patients (*n* = 20) and healthy subjects (*n* = 19) in October-April; and (**C**) patients (*n* = 11) and healthy subjects (*n* = 15) in May-September. Data are the means (long horizontal lines) ± SD. ***, *p* < 0.001; ****, *p* < 0.0001.

**Figure 2:**
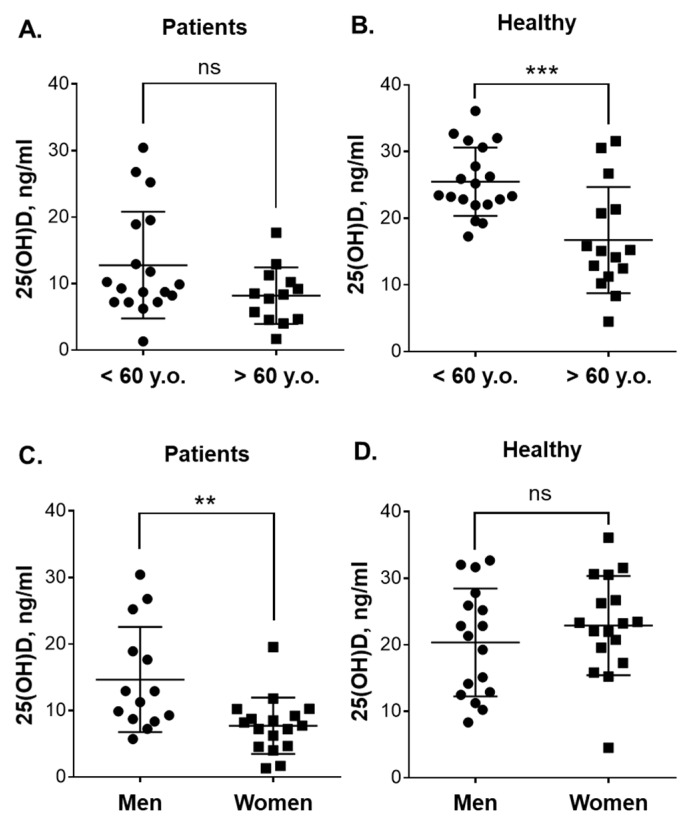
Plasma 25(OH)D levels in blood samples of patients with leukemia and healthy subjects of different age and gender. Comparison between: (**A**) younger (*n* = 18) and older (*n* = 13) patients; (**B**) younger (*n* = 19) and older (*n* = 15) healthy subjects, (**C**) male (*n* = 14) and female (*n* = 17) patients; and (**D**) male (*n* = 17) and female (*n* = 17) healthy subjects. Data are the means (long horizontal lines) ± SD. **, *p* < 0.01; ***, *p* < 0.001; ns, not significant; y.o., years old.

All measurements in the 25(OH)D and VDR assays were performed in triplicate. The significance of the differences between the means was assessed by nonparametric Mann–Whitney test. Overall survival curves were generated by Kaplan–Meier analysis. A log-rank (Mantel–Cox) test was used to evaluate the significance of differences between participant groups. *P* < 0.05 was considered statistically significant. The above statistical analyses were carried out by the GraphPad Prism 6.0 program (Graph-Pad Software, San Diego, CA, USA). Power analysis was performed by G*Power software (Version 3.1.9.4, Heinrich Heine Universität, Düsseldorf, Germany), as described previously [21]. Power was computed using post-hoc test based on Mann–Whitney test results and given α (0.05), sample size and effect size. Pooled SD values (σ) were calculated by Microsoft Excel. Power computations are provided in Supplementary Materials. Although high statistical power (0.84–0.99) was obtained for all the comparisons showing statistically significant differences (Figure 1A–C; Figure 2B,C and Figure S1A,B), we are aware that due to a relatively small sample size of the participant (sub)groups these power values may be overestimated. 

## 3. Results

A total of 31 patients (14 men and 17 women) from Karaganda city and Karaganda region were included in this study (Table 1). The median age was 57 years (range: 28–85). Most of these enrolled patients (twenty-five; 80.6%) were those with newly diagnosed CML in the chronic phase (<10% blasts in the bone marrow) who were referred to the DNA Diagnostics Laboratory (the Dippner Health Center, Karaganda) for the RT-qPCR detection of the chimeric *BCR-ABL* transcript [22] in isolated PBMCs. These patients had an appreciable myeloid hyperplasia in the bone marrow as well as substantial leukocytosis (>100 × 10^9^ WBC/L) with neutrophilia. The diagnosis was confirmed by the presence of the *BCR-ABL* transcript (Table 1). Four patients had newly diagnosed AML with high percentages of leukemic blasts (45–52%) in the hypocellular bone marrow; and the other two patients had either CLL or ALL (Table 1). For the control group, we selected demographically similar 34 adult residents of the Karaganda region (17 men and 17 women) who did not suffer from chronic diseases or hereditary pathologies (Table 2). The median age of this group was 54 years (range: 26–78). While about 79% of male patients and 56% of male volunteers were current smokers, 16 out of 17 female patients and all 18 healthy women were nonsmokers. There were 48.4% Asians and 51.6% Caucasians in the patient group and 58.8% Asians and 41.2% Caucasians in the healthy volunteer group (Table 1 and Table 2). 

The diet history of the participants was not recorded except that it was ascertained that they did not use vitamin D-containing supplements. In addition, overall health assessment did not reveal signs of undernourishment. Body mass index (BMI; kg/m^2^) distribution (Table 1 and Table 2) was found to be very similar among the patients (26.1 ± 5.2; 95% CI 24.2–28.0; *n* = 31) and the healthy volunteers (26.4 ± 4.5; 95% CI 24.8–27.9; *n* = 34). Thus, we generally assumed that the participants consumed foods that are most common in Kazakhstan, i.e., those based on meat, flour and dairy products [17,23] and known to be high in saturated fat, free sugars and salt (HFSS foods) [*http://www.euro.who.int/__data/assets/pdf_file/0010/396190/WHO-Nutrition-Kazakhtsan-EN.pdf?ua=1*].

As shown in Figure 1A, the average plasma 25(OH)D concentration in leukemia patients (10.8 ± 7.0 ng/mL; 95% CI 8.3–13.4; *n* = 31) was considerably lower (*p* < 0.0001) compared to that in healthy subjects (21.6 ± 7.8 ng/mL; 95% CI 18.9–24.3; *n* = 34). When analyzed separately, CML patients and the small group of non-CML patients (Table 1) demonstrated similar reduced vitamin D levels (10.3 ± 6.3 ng/mL; *n* = 25 vs. 13.3 ± 9.8 ng/mL; *n* = 6). We then compared 25(OH)D concentrations in the blood samples from patients and healthy individuals collected during the colder season (October-April) and the warmer season (May-September) in the Karaganda region (https://weatherspark.com/y/107797/Average-Weather-in-Karagandy-Kazakhstan-Year-Round). The comparison showed no significant seasonal variation in vitamin D status within the same participant groups, i.e., 10.84 ± 7.69 ng/mL; 95% CI 7.24–14.43 (*n* = 20) vs. 10.87 ± 5.90 ng/mL; 95% CI 6.91–14.84 (*n* = 11) for the patients and 21.44 ± 8.87 ng/mL; 95% CI 17.17–25.72 (*n* = 19) vs. 21.83 ± 6.43 ng/mL; 95% CI 18.27–25.39 (*n* = 15) for the healthy subjects during October-April vs. May-September, respectively. However, similar to the data shown in Figure 1A, marked significant differences were observed when 25(OH)D levels were compared between the patients and healthy controls during both the colder (Figure 1B; *p* = 0.0003) and the warmer (Figure 1C; *p* = 0.0002) seasons. 

Since the median age of patients and healthy volunteers was close to 60, the participants of <60 years were considered the “younger” group and those of >60 years the “older” group. No significant difference in 25(OH)D levels was observed between the younger (*n* = 18) and older (*n* = 13) patients (Figure 2A). On the other hand, as shown in Figure 2B, the younger healthy adults had significantly higher (*p* = 0.0007) concentrations of this vitamin D derivative (25.5 ± 5.1 ng/mL; 95% CI 23.0–27.9; *n* = 19) than the older ones (16.7 ± 7.9 ng/mL; 95% CI 12.3–21.1; *n* = 15). Comparison of the vitamin D status in younger patients vs. younger healthy participants and in older patients vs. older healthy participants revealed significantly lower 25(OH)D plasma concentrations in patients of both age groups (Figure S1). When gender-related differences were examined (Figure 2C), it was found that the female patients had significantly lower 25(OH)D concentrations (7.7 ± 4.2 ng/mL; 95% CI 5.5–9.9; *n* = 17) than the male patients (14.7 ± 7.9 ng/mL; 95% CI 10.1–19.2; *n* = 14; *p* = 0.004). However, there was no significant differences in plasma 25(OH)D levels between healthy men and women (Figure 2D).

**Figure 3:**
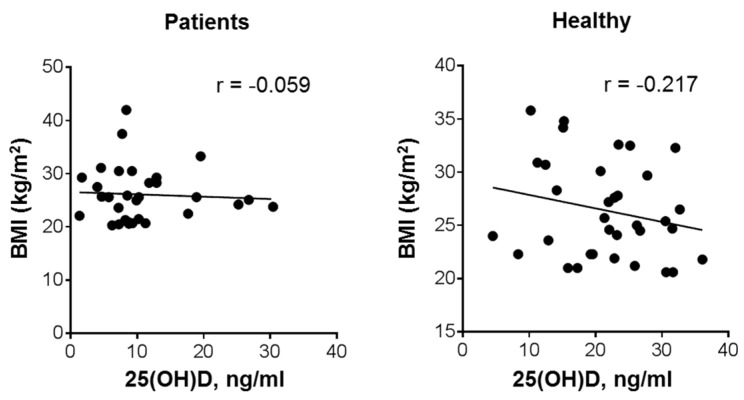
Relationship between plasma 25(OH)D levels and BMI in patients with leukemia and healthy subjects. Bivariate (Pearson) correlation analysis (31 patients and 34 healthy volunteers); r, correlation coefficient.

As shown in Figure 3, there was practically no correlation between BMI and 25(OH)D blood levels in the patient group, though the volunteer group demonstrated a weak inverse relationship between these parameters (r = −0.217; *p* = 0.22). A similar, though statistically significant, inverse relationship has been reported in previous studies involving larger cohorts of healthy subjects [24,25,26]. 

Consistent with the data showing lower 25(OH)D levels in patients than in healthy volunteers in general (Figure 1), Asian patients had a pronounced vitamin D deficiency (10.71 ± 5.93 ng/mL; 95% CI 7.43–14.00; *n* = 15) compared to Asian volunteers *(*23.21 ± 6.70 ng/mL; 95% CI 20.07–26.35; *n* = 20; *p* < 0.0001). Likewise, Caucasian patients had reduced 25(OH)D concentrations (10.98 ± 8.07 ng/mL; 95% CI 6.67–5.28; *n* = 16) compared to a borderline-normal vitamin D status in healthy Caucasian subjects (19.33 ± 8.85 ng/mL; 95% CI 14.22–24.44; *n* = 14; *p* = 0.012). Otherwise, there was no significant difference in circulating 25(OH)D levels between Asians and Caucasians within each of the two participating cohorts, i.e., patients or volunteers (see above). 

We did not observe a significant influence of tobacco use on vitamin D status. For instance, nonsmoking and smoking male volunteers had comparable 25(OH)D levels—21.60 ± 8.65 ng/mL; 95% CI 13.60–29.60 (*n* = 7) and 19.10 ± 8.47 ng/mL; 95% CI 12.59–25.61 (*n* = 9), respectively. Similar, though slightly lower, 25(OH)D concentrations were measured in smoking male patients (16.86 ± 7.55 ng/mL; 95% CI 11.79–21.93; *n* = 11). On the other hand, 16 out of 17 female patients who were nonsmokers had the lowest 25(OH)D levels among all the participants (Table 1 and Table 2; Figure 2C), whereas healthy nonsmoking women had fairly normal 25(OH)D concentrations, which were even slightly higher than those found in healthy men (Table 2; Figure 2D).

**Figure 4:**
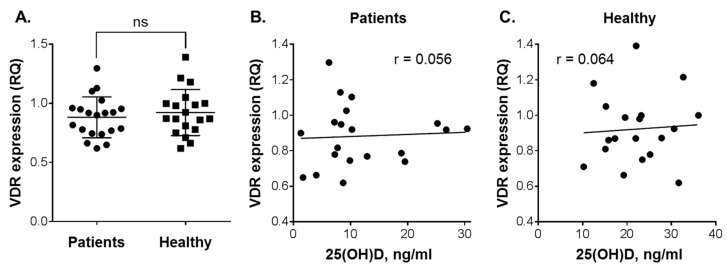
Vitamin D receptor (VDR) expression in peripheral blood mononuclear cells (PBMC) vs. 25(OH)D levels in the plasma of patients with leukemia and healthy subjects. (**A**) Comparison of VDR expression between patients (*n* = 20) and healthy individuals (*n* = 19). Data are the means (long horizontal lines) ± SD. Correlation analysis of VDR expression and 25(OH)D levels in patients (**B**) and healthy subjects (**C**); ns, not significant; r, correlation coefficient.

Vitamin D intervention studies have demonstrated that elevation of circulating 25(OH)D levels results in increased VDR transcriptional activity in PBMCs, which are the easiest available vitamin D-responsive primary human cell types [27,28]. Furthermore, it has been shown that VDR expression in white blood cells can be dose-dependently upregulated by vitamin D derivatives [29,30]. To examine whether plasma vitamin D concentrations of our study participants would correlate with their cellular VDR expression, we measured VDR mRNA levels in PBMCs isolated from those individuals who consented to the latter assay. Using one of the healthy subject’s cDNA sample as a calibrator (see Materials and Methods), we found very similar VDR expression levels in patients with leukemia and healthy individuals (Figure 4A). Furthermore, no correlation between VDR expression and plasma 25(OH)D levels was observed in either the patients (r = 0.056; Figure 4B) or the healthy subjects (r = 0.064; Figure 4C) that participated in this study. 

**Figure 5:**
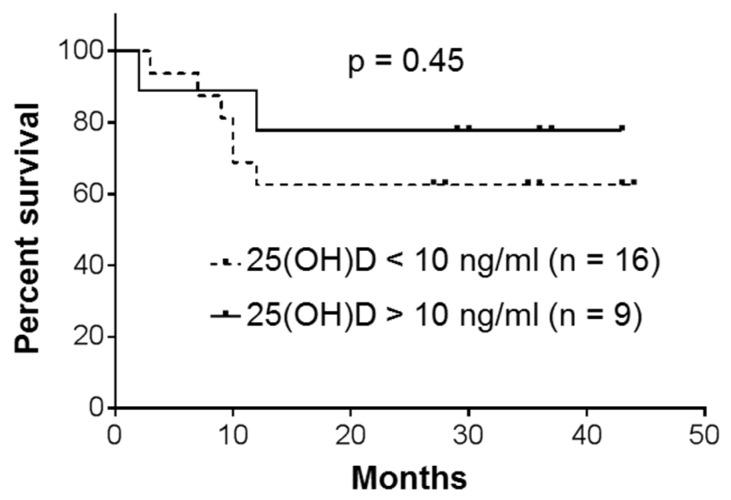
Overall survival of patients with chronic myeloid leukemia (CML). Kaplan–Meier analysis with a log-rank (Mantel–Cox) test.

The patients were followed up till November, 2019, or death. All the patients with acute leukemias (*n* = 5; Table 1) died within 1–3 months. Overall survival curves for patients with CML, the largest group of the participants, are presented in Figure 5. The data demonstrate a somewhat better survival rate for the patients whose plasma 25(OH)D levels were found to be >10 ng/mL (*n* = 9) compared to those with lower 25(OH)D levels (<10 ng/mL; *n* = 16). However, the difference between the two CML subsets was not significant (*p* = 0.45), most likely due to limited numbers of participants. Furthermore, comparison between the patients with all types of leukemia showed practically the same low plasma 25(OH)D concentrations for both survivors and nonsurvivors (10.81 ± 6.64 ng/mL; 95% CI 7.50–14.11; *n* = 18 vs. 10.91 ± 7.75 ng/mL; 95% CI 6.23–15.59; *n* = 13). Although relative VDR expression in PBMCs varied among the tested patients, the survivors did not present significantly higher expression levels compared to the nonsurvivors (0.91 ± 0.18; 95% CI 0.79–1.02; *n* = 13 vs. 0.84 ± 0.16; 95% CI 0.70–0.98; *n* = 7; *p* = 0.34; Table 1). 

## 4. Discussion

The major finding of the present small-scale single-center pilot study conducted in Kazakhstan for the first time is that the participating adult patients with leukemia, who were admitted to the Karaganda Regional Clinical Hospital, had substantially lower plasma levels of vitamin D (measured as 25(OH)D) compared to a demographically similar control group of adult healthy residents of the same region. Moreover, the majority of the patients, especially the female ones, had severe vitamin D deficiency (<10–12 ng/mL), in accordance with the international consensus [6]. On the other hand, we did not observe a significant influence of age, BMI, smoking habits or ethnicity on the patient vitamin D status. Interestingly, although it has been widely reported that there is a seasonal pattern of vitamin D status in populations, particularly in temperate regions of the world (e.g., [31,32]), we also did not observe seasonal variations of 25(OH)D levels in either the patient or the healthy control group. This finding indicates that the difference in vitamin D status between the patients and healthy subjects who participated in this study is not related to seasonality. The lack of seasonal 25(OH)D changes in vitamin D-deficient patients with cancer [33] and other diseases (e.g. [34,35,36]) has been previously reported. There may be several reasons for such a phenomenon, including limited outdoor activity, low dietary intake of vitamin D and higher rates of 25(OH)D metabolism in patients which may affect its residence time in the blood. The fact that seasonal changes in 25(OH)D were not detected in our healthy control group is puzzling and needs to be re-estimated in a larger and more detailed study.

Low vitamin D levels are frequently associated with an increased risk and worse clinical outcome of cancers (e.g. [37,38]), including hematological malignancies [13,14]. Thus, our data are consistent with those previously reported by other research groups for patients with different types of leukemia. For instance, Lee et al. [39] have shown that serum 25(OH)D levels in 30% of 105 patients with newly diagnosed AML were below 20 ng/mL (defined by the authors as vitamin D deficiency), which was associated with worse relapse-free survival compared to those patients who had normal vitamin D status. Similar low levels of 25(OH)D were detected by Thomas et al. [40] in many patients with various hematological diseases (acute and chronic leukemias, chronic lymphoid disorders, etc.). This was also inversely correlated with the response to therapy. 

Although the above and other published studies related to blood cancers (e.g. [13]) did not include control groups of healthy individuals, it was reported that patients with nonmalignant hematological disorders, such as autoimmune hemolytic anemia, immune thrombocytopenic purpura or chronic idiopathic neutropenia, had decreased 25(OH)D levels compared to healthy individuals [41]. Several reports demonstrated the lack of correlation between vitamin D levels and age of patients or healthy individuals (e.g. [30,40]); however, we observed an inverse correlation in healthy subjects, i.e., the younger adults (>60 years old) had significantly higher plasma 25(OH)D levels (25.5 ± 5.1 ng/mL) than the older ones (16.7 ± 7.9 ng/mL). 

The role of VDR in carcinogenesis and outcomes of hematopoietic malignancies remains poorly studied, though high expression of VDR has been described in several lymphoid cancers [42,43,44]. In the present study, we found no significant differences in VDR expression between patients and healthy volunteers and a lack of correlation between VDR expression and plasma 25(OH)D levels. In addition, there was no correlation between the age of patients and the VDR mRNA levels in their cells (r = −0.29), which is similar to the data obtained by Coleman et al. [30] in healthy donors (r = −0.23). Further, relative VDR expression in PBMCs of the patients who were alive at the end of the follow-up period did not significantly differ from that in the nonsurvivors. More studies are needed to determine a possible association between VDR levels and the risk of leukemia development and/or disease prognosis. 

Due to the lack of large population-based vitamin D studies in Kazakhstan, it is unclear if high incidence rates of leukemia in the Karaganda region observed during 2003–2013 [18] are related to the prevalence of vitamin D deficiency in Central Kazakhstan [17]. Leukemia incidence in this area may be influenced by multiple important factors, including its proximity to the former Semipalatinsk nuclear test site and high levels of toxic air pollutants [45]. Although our present pilot study is observational and involves small numbers of participants, it provides initial findings that warrant a larger and more detailed investigation into the possible contribution of vitamin D deficiency to high incidence of leukemia in Central Kazakhstan and other regions of the country.

Causality between vitamin D deficiency and the development, progression or curability of cancer/leukemia is hard to prove and remains a matter of debate (see [38,46] for recent reviews). Observational studies are generally unable to determine whether vitamin D is causally associated with cancer incidence or mortality, whereas previous small-scale randomized controlled trials (RCTs) of vitamin D supplementation and cancer incidence and mortality produced inconsistent results. Several recent large-scale long-term RCTs, such as VITAL [47] and ViDA [48], initially showed that supplementation with vitamin D did not result in a lower incidence of invasive cancer. However, updated secondary analyses of VITAL and other RCTs revealed that supplementation significantly reduced not only total cancer mortality [49] but also total cancer incidence in certain participant subgroups [38,47]. Importantly, a secondary analysis of data from the Women’s Health Initiative Calcium/Vitamin D (CaD) has also shown protective associations between calcium/vitamin D supplementation and risk of hematopoietic malignancies in older women [50]. Only few studies investigated the effect of postdiagnosis supplementation with vitamin D on survival of patients with cancer. For instance, a large study conducted in Ireland demonstrated that supplementation was associated with a reduction in breast cancer-specific mortality [51]. In another study, treatment with 25(OH)D combined with the iron chelator deferasirox resulted in an increase in median survival of elderly patients with AML compared to those receiving best supportive care [52].

One limitation of VITAL and ViDA was that the mean baseline 25(OH)D concentrations of the participants were in the normal range, e.g., 30.8 ± 10 ng/mL for VITAL [47]. Therefore, it is likely that if conducted in countries such as Kazakhstan, with “naturally” vitamin D deficient/insufficient populations, similar long-term RCTs and studies of de novo vitamin D supplementation in postdiagnosis patients would have much greater power in evaluating the role of vitamin D in cancer/leukemia risk, progression and mortality. 

## 5. Conclusions

Our pilot study demonstrated that in Central Kazakhstan, the majority of patients with leukemia, particularly females, had severe vitamin D deficiency while the matched healthy control group showed a borderline sufficient vitamin D status. These findings warrant further investigation of the role of vitamin D deficiency in high incidence of leukemia in Kazakhstan. We believe that countries such as Kazakhstan, with “naturally” vitamin D deficient/insufficient populations may represent ideal sites for long-term RCTs of vitamin D supplementation in healthy individuals and patients with neoplasia to elucidate the role of vitamin D in cancer risk, progression and mortality. 

## Figures and Tables

**Table 1:** Characteristics of participating patients (*n* = 31).

No.	Sex	Age	Ethnicity	BMI ^a^	Tobacco Use	25(OH)D ng/mL	25(OH)D Test Date	VDR ^b^ (RQ) ^c^	Diagnosis	Clinical/Laboratory Data	Treatment	Survival (Months) ^j^
1	F	40	Caucasian	22.1	No	1.31	March 2016	0.90	CML ^e^	Bcr-Abl transcript; chronic phase; myeloid hyperplasia, leukocytosis (>100 × 10^9^ WBC ^i^/L)	Nilotinib	LTS ^k^
2	F	78	Asian	29.3	No	1.67	March 2016	0.65	CML	Bcr-Abl transcript; chronic phase; myeloid hyperplasia, leukocytosis (>100 × 10^9^ WBC/L)	Nilotinib	10
3	F	61	Caucasian	27.5	No	4.00	March 2016	0.66	CML	Bcr-Abl transcript; chronic phase; myeloid hyperplasia, leukocytosis (>100 × 10^9^ WBC/L)	Nilotinib	LTS
4	F	78	Asian	31.1	No	4.55	June 2017	ND ^d^	CML	Bcr-Abl transcript; chronic phase; myeloid hyperplasia, leukocytosis (>100 × 10^9^ WBC/L)	Imatinib	10
5	M	85	Caucasian	25.6	No	5.72	June 2017	ND	CML	Bcr-Abl transcript; chronic phase; myeloid hyperplasia, leukocytosis (>100 × 10^9^ WBC/L)	Imatinib	3
6	F	48	Asian	20.3	No	6.24	February 2016	1.30	CML	Bcr-Abl transcript; chronic phase; myeloid hyperplasia, leukocytosis (>100 × 10^9^ WBC/L)	Nilotinib	LTS
7	F	46	Caucasian	23.6	No	7.19	March 2016	0.96	CML	Bcr-Abl transcript; chronic phase; myeloid hyperplasia, leukocytosis (>100 × 10^9^ WBC/L)	Nilotinib	LTS
8	F	53	Caucasian	20.5	No	7.24	June 2017	0.78	CML	Bcr-Abl transcript; chronic phase; myeloid hyperplasia, leukocytosis (>100 × 10^9^ WBC/L)	Imatinib	LTS
9	F	39	Caucasian	21.3	No	8.22	May 2017	1.13	CML	Bcr-Abl transcript; chronic phase; myeloid hyperplasia, leukocytosis (>100 × 10^9^ WBC/L)	Imatinib	LTS
10	F	65	Caucasian	42.0	Yes	8.36	October 2016	0.95	CML	Bcr-Abl transcript; chronic phase; myeloid hyperplasia, leukocytosis (>100 × 10^9^ WBC/L)	Imatinib	9
11	F	68	Asian	25.9	No	8.51	June 2017	ND	CML	Bcr-Abl transcript; chronic phase; myeloid hyperplasia, leukocytosis (>100 × 10^9^ WBC/L)	Nilotinib	12
12	M	28	Caucasian	20.9	No	8.72	November 2016	0.62	CML	Bcr-Abl transcript; chronic phase; myeloid hyperplasia, leukocytosis (>100 × 10^9^ WBC/L)	Imatinib	LTS
13	F	53	Asian	20.6	No	8.74	December 2016	ND	CML	Bcr-Abl transcript; chronic phase; myeloid hyperplasia, leukocytosis (>100 × 10^9^ WBC/L)	Nilotinib	LTS
14	F	62	Asian	30.5	No	9.20	June 2017	ND	CML	Bcr-Abl transcript; chronic phase; myeloid hyperplasia, leukocytosis (>100 × 10^9^ WBC/L)	Imatinib	LTS
15	M	56	Asian	20.7	Yes	9.27	February 2016	1.03	CML	Bcr-Abl transcript; chronic phase; myeloid hyperplasia, leukocytosis (>100 × 10^9^ WBC/L)	Hydroxyurea	LTS
16	M	57	Caucasian	25.0	Yes	9.89	April 2016	0.75	CML	Bcr-Abl transcript; chronic phase; myeloid hyperplasia, leukocytosis (>100 × 10^9^ WBC/L)	Imatinib	7
17	M	39	Asian	25.6	No	10.25	June 2017	0.92	CML	Bcr-Abl transcript; chronic phase; myeloid hyperplasia, leukocytosis (>100 × 10^9^ WBC/L)	Nilotinib	LTS
18	M	65	Asian	20.7	Yes	11.25	May 2017	ND	CML	Bcr-Abl transcript; chronic phase; myeloid hyperplasia, leukocytosis (>100 × 10^9^ WBC/L)	Imatinib	LTS
19	F	53	Asian	28.3	No	11.79	June 2017	ND	CML	Bcr-Abl transcript; chronic phase; myeloid hyperplasia, leukocytosis (>100 × 10^9^ WBC/L)	Nilotinib	LTS
20	M	72	Caucasian	28.3	Yes	12.92	February 2016	0.77	CML	Bcr-Abl transcript; chronic phase; myeloid hyperplasia, leukocytosis (>100 × 10^9^ WBC/L)	Imatinib	12
21	M	58	Caucasian	29.3	Yes	12.92	November 2016	ND	CML	Bcr-Abl transcript; chronic phase; myeloid hyperplasia, leukocytosis (>100 × 10^9^ WBC/L)	Nilotinib	LTS
22	M	76	Asian	22.5	Yes	17.65	May 2017	ND	CML	Bcr-Abl transcript; chronic phase; myeloid hyperplasia, leukocytosis (>100 × 10^9^ WBC/L)	Nilotinib	1
23	M	42	Asian	25.6	Yes	18.91	April 2016	0.79	CML	Bcr-Abl transcript; chronic phase; myeloid hyperplasia, leukocytosis (>100 × 10^9^ WBC/L)	Imatinib	LTS
24	M	43	Asian	24.2	Yes	25.22	June 2017	0.95	CML	Bcr-Abl transcript; chronic phase; myeloid hyperplasia, leukocytosis (>100 × 10^9^ WBC/L)	Nilotinib	LTS
25	M	43	Caucasian	25.1	Yes	26.78	October 2016	0.92	CML	Bcr-Abl transcript; chronic phase; myeloid hyperplasia, leukocytosis (>100 × 10^9^ WBC/L)	Imatinib	LTS
26	F	73	Caucasian	25.7	No	4.66	December 2016	ND	AML ^f^	Low bone marrow (BM) cellularity; ~52% blasts in the BM; myelosuppression	Cytarabine + doxorubicin	1
27	F	68	Asian	21.5	No	10.22	November 2016	1.10	AML	Low BM cellularity; ~50% blasts in the BM; myelosuppression; breast cancer 2 years before AML	Cytarabine + doxorubicin	3
28	F	54	Caucasian	33.3	No	19.53	February 2016	0.74	AML	Low BM cellularity; ~45% blasts in the BM; myelosuppression. M0 subtype with CD7 co-expression	Cytarabine + doxorubicin	3
29	M	59	Caucasian	23.8	Yes	30.43	December 2016	0.92	AML	Low BM cellularity; ~51% blasts in the BM; myelosuppression	Cytarabine + doxorubicin	3
30	M	59	Asian	30.5	Yes	7.24	March 2016	ND	CLL ^g^	B-cell CLL (CD19^+^/CD5^+^/CD23^−^/CD200^+^/CD22^+^/CD20^+^/CD43^+^/CD38^+^/CD79b^−^/CD10^−^/FMC7^−^); leukocytosis (17,000 WBC/µl) with absolute lymphocytosis; ~2% prolymphocytes and 9% atypical lymphocytes in the blood; moderate BM cellularity.	Imatinib	LTS
31	F	68	Caucasian	37.5	No	7.74	March 2016	0.82	ALL ^h^	Pre-B (BIII) ALL (cytIgM^+^/sIgM^−^/CD19^+^/CD10^+^/CD34^+^/HLADR^+^/cytCD22^+^/ sCD22^dim^/CD20^dim^/CD7^−^/CD13^−^/CD33^−^/MPO^−^); ~44% blasts in the BM; low BM cellularity.	Imatinib	1

^a^ BMI, body mass index; ^b^ VDR, vitamin D receptor; ^c^ RQ, relative quantification; ^d^ ND, not determined; ^e^ CML, chronic myeloid leukemia; ^f^ AML, acute myeloid leukemia; ^g^ CLL, chronic lymphocytic leukemia; ^h^ ALL, acute lymphoblastic leukemia; ^i^ WBC, white blood cells; ^j^ Survival since the start of treatment; ^k^ LTS, long-term survivor.

**Table 2:** Characteristics of healthy volunteers (*n* = 34).

	Men									Women			
No.	Age	Ethnicity	BMI	Tobacco Use	25(OH)D ng/mL	25(OH)D Test Date	No.	Age	Ethnicity	BMI	Tobacco Use	25(OH)D ng/mL	25(OH)D Test Date
1	71	Caucasian	22.3	No	8.34	October 2017	18	78	Caucasian	24.0	No	4.52	December 2016
2	74	Caucasian	35.8	Yes	10.23	March 2016	19	71	Caucasian	34.8	No	15.23	June 2017
3	68	Asian	30.9	Yes	11.24	April 2017	20	70	Caucasian	21.0	No	15.84	May 2016
4	68	Asian	30.7	Yes	12.46	June 2017	21	26	Caucasian	21.0	No	17.26	July 2016
5	65	Asian	23.6	Yes	12.88	May 2016	22	55	Asian	22.3	No	19.56	May 2016
6	60	Caucasian	28.3	Yes	14.15	February 2016	23	64	Caucasian	30.1	No	20.75	February 2017
7	63	Asian	34.2	No	15.11	November 2016	24	42	Asian	27.2	No	21.95	October 2017
8	48	Asian	22.3	No	19.26	May 2017	25	33	Asian	24.6	No	22.05	November 2016
9	77	Caucasian	25.7	No	21.34	April 2016	26	35	Caucasian	24.1	No	23.21	November 2017
10	52	Asian	21.9	No	22.83	October 2016	27	36	Asian	27.8	No	23.33	September 2016
11	48	Asian	27.6	No	22.84	May 2016	28	36	Asian	32.6	No	23.45	June 2017
12	32	Caucasian	32.5	Yes	25.20	April 2017	29	52	Asian	25.0	No	26.23	June 2016
13	48	Asian	21.2	Yes	25.90	June 2016	30	63	Asian	24.5	No	26.70	March 2017
14	53	Caucasian	29.7	Yes	27.80	October 2016	31	68	Asian	25.4	No	30.53	June 2017
15	58	Asian	20.6	No	31.66	April 2016	32	53	Caucasian	20.6	No	30.63	June 2016
16	41	Asian	32.3	Yes	32.02	June 2016	33	75	Asian	24.7	No	31.56	April 2016
17	48	Asian	26.5	No	32.68	October 2016	34	30	Caucasian	21.8	No	36.10	March 2016

## Supplementary Materials

The following are available online at https://www.mdpi.com/2072-6643/12/5/1229/s1, Figure S1: Plasma 25(OH)D levels in younger and older patients with leukemia and healthy subjects.
