# Supplementary material for: Plasma 25-Hydroxyvitamin D Levels and VDR Gene Expression in Peripheral Blood Mononuclear Cells of Leukemia Patients and Healthy Subjects in Central Kazakhstan
Source: Nutrients. 2020 Apr 26;12(5):1229. doi: 10.3390/nu12051229 (PMC7281978; doi:10.3390/nu12051229)
Supplement: Supplementary file 1 [file nutrients-12-01229-s001.pdf]

nutrients

MDPI

Article

Plasma 25-hydroxyvitamin D levels and VDR gene expression in peripheral blood mononuclear cells of leukemia patients and healthy subjects in Central Kazakhstan

Assel G. Zhumina [1], Constantin G. Li [2] Anna A. Konovalova [1,2], Yelena A. Li [2], Margarita Yu. Ishmuratova [1], Gayane P. Pogossyan [1,2] and Michael Danilenko [3,*]

[1] Department of Botany, Academician Y.A. Buketov Karaganda State University, Karaganda 100028, Kazakhstan; asbiol@list.ru (A.G.Z.); anjuta.kon_1986@mail.ru (A.A.K.); margarita.ishmur@mail.ru (M.Y.I.); gayane_63@mail.ru (G.P.P.)

[2] DNA Diagnostics Laboratory, the Dippner Health Center, Karaganda 100009, Kazakhstan; lee11@mail.ru (C.G.L.); helennala@gmail.com (Y.A.L.)

[3] Department of Clinical Biochemistry and Pharmacology, Faculty of Health Sciences, Ben-Gurion University of the Negev, Beer-Sheva 84105, Israel. misha@bgu.ac.il (M.D.)

* Correspondence: misha@bgu.ac.il; Tel.: +972-8647- 9969 (M.D.)

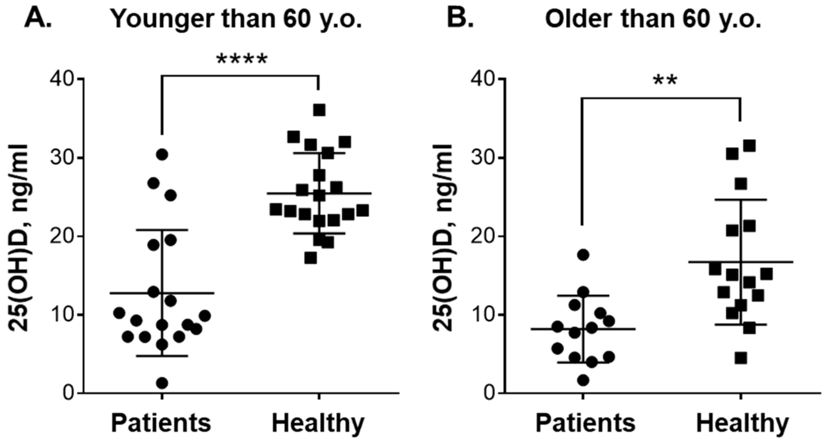

Supplementary Figure S1. Plasma 25(OH)D levels in younger and older patients with leukemia and healthy subjects. Comparison between: (**A**) younger patients (n = 18) and younger healthy subjects (n = 19) and (**B**) older patients (n = 13) and older healthy subjects (n = 15). Data are the means (long horizontal lines) ± SD. **, P = 0.0018; ****, P < 0.0001; y.o., years old.

Power analysis of the data shown in Fig. 1A

t tests – Means: Wilcoxon–Mann–Whitney test (two groups)
Options:A.R.E. method

**Analysis:**			Post hoc: Compute achieved power
**Input:**	Tail(s)	=	Two
	Parent distribution	=	Normal
	Effect size d	=	1.4501348
	α err prob	=	0.05
	Sample size group 1	=	31
	Sample size group 2	=	34
**Output:**	Noncentrality parameter δ	=	5.7063406
	Critical t	=	2.0002496
	Df	=	60.0704278
	Power (1–β err prob)	=	0.9998705

Power analysis of the data shown in the new Fig. 1B

t tests – Means: Wilcoxon–Mann–Whitney test (two groups)
Options:A.R.E. method

**Analysis:**			Post hoc: Compute achieved power
**Input:**	Tail(s)	=	Two
	Parent distribution	=	Normal
	Effect size d	=	1.2786490
	α err prob	=	0.05
	Sample size group 1	=	20
	Sample size group 2	=	19
**Output:**	Noncentrality parameter δ	=	3.9002867
	Critical t	=	2.0296092
	Df	=	35.2422567
	Power (1–β err prob)	=	0.9665239

Power analysis of the data shown in the new Fig. 1C

t tests – Means: Wilcoxon–Mann–Whitney test (two groups)
Options:A.R.E. method

**Analysis:**			Post hoc: Compute achieved power
**Input:**	Tail(s)	=	Two
	Parent distribution	=	Normal
	Effect size d	=	1.7620579
	α err prob	=	0.05
	Sample size group 1	=	11
	Sample size group 2	=	15
**Output:**	Noncentrality parameter δ	=	4.3377165
	Critical t	=	2.0695195
	Df	=	22.8281711
	Power (1–β err prob)	=	0.9857007

Power analysis of the data shown in the new Fig. 2B

t tests – Means: Wilcoxon-Mann-Whitney test (two groups)
Options:A.R.E. method

**Analysis:**			Post hoc: Compute achieved power
**Input:**	Tail(s)	=	Two
	Parent distribution	=	Normal
	Effect size d	=	1.4135703
	α err prob	=	0.05
	Sample size group 1	=	19
	Sample size group 2	=	15
**Output:**	Noncentrality parameter δ	=	3.9993193
	Critical t	=	2.0409589
	Df	=	30.4676084
	Power (1−β err prob)	=	0.9719585

Power analysis of the data shown in the new Fig. 2C

t tests – Means: Wilcoxon-Mann-Whitney test (two groups)
Options:A.R.E. method

**Analysis:**			Post hoc: Compute achieved power
**Input:**	Tail(s)	=	Two
	Parent distribution	=	Normal
	Effect size d	=	1.1282468
	α err prob	=	0.05
	Sample size group 1	=	14
	Sample size group 2	=	17
**Output:**	Noncentrality parameter δ	=	3.0549031
	Critical t	=	2.0497359
	Df	=	27.6028194
	Power (1−β err prob)	=	0.8382074

Power analysis of the data shown in Supplementary Fig. S1A

t tests – Means: Wilcoxon-Mann-Whitney test (two groups)
Options:A.R.E. method

**Analysis:**			Post hoc: Compute achieved power
**Input:**	Tail(s)	=	Two
	Parent distribution	=	Normal
	Effect size d	=	1.8983558
	α err prob	=	0.05
	Sample size group 1	=	18
	Sample size group 2	=	19
**Output:**	Noncentrality parameter δ	=	5.6399531
	Critical t	=	2.0337448
	Df	=	33.3323974
	Power (1−β err prob)	=	0.9997772

Power analysis of the data shown in Supplementary Fig. S1B

t tests – Means: Wilcoxon-Mann-Whitney test (two groups)
Options: A.R.E. method

**Analysis:**			Post hoc: Compute achieved power
**Input:**	Tail(s)	=	Two
	Parent distribution	=	Normal
	Effect size d	=	1.3104455
	α err prob	=	0.05
	Sample size group 1	=	13
	Sample size group 2	=	15
**Output:**	Noncentrality parameter δ	=	3.3794260
	Critical t	=	2.0606450
	Df	=	24.7380304
	Power (1−β err prob)	=	0.9007164

© 2019 by the authors. Submitted for possible open access publication under the terms and conditions of the Creative Commons Attribution (CC BY) license (http://creativecommons.org/licenses/by/4.0/).
